# Bioactivities of EF24, a Novel Curcumin Analog: A Review

**DOI:** 10.3389/fonc.2018.00614

**Published:** 2018-12-11

**Authors:** Yonghan He, Wen Li, Guangrong Hu, Hui Sun, Qingpeng Kong

**Affiliations:** ^1^State Key Laboratory of Genetic Resources and Evolution, Kunming Institute of Zoology, The Chinese Academy of Sciences, Kunming, China; ^2^Department of Endocrinology, The Third People's Hospital of Yunnan Province, Kunming, China; ^3^Department of Emergency, The Second Affiliated Hospital of Harbin Medical University, Harbin, China

**Keywords:** curcumin, EF24, cancer, bio-activity, inflammation, mechanism

## Abstract

Curcumin is an attractive agent due to its multiple bioactivities. However, the low oral bioavailability and efficacy profile hinders its clinical application. To improve the bioavailability, many analogs of curcumin have been developed, among which EF24 is an excellent representative. EF24 has enhanced bioavailability over curcumin and shows more potent bioactivity, including anti-cancer, anti-inflammatory, and anti-bacterial. EF24 inhibits tumor growth by inducing cell cycle arrest and apoptosis, mainly through its inhibitory effect on the nuclear factor kappa B (NF-κB) pathway and by regulating key genes through microRNA (miRNA) or the proteosomal pathway. Based on the current structure, more potent EF24 analogs have been designed and synthesized. However, some roles of EF24 remain unclear, such as whether it induces or inhibits reactive oxygen species (ROS) production and whether it stimulates or inhibits the mitogen activated kinase-like protein (MAPK) pathway. This review summarizes the known biological and pharmacological activities and mechanisms of action of EF24.

## Introduction

Many natural products have been identified for various medicinal purposes (1–3). Curcumin, a hydrophobic polyphenol derived from the rhizome of the herb *Curcuma longa* is a well-defined example. It has demonstrated wide-spectrum biological and pharmacological activities, such as antioxidant (4, 5), anti-inflammatory (6) antimicrobial (7–10), and anti-cancer (11) activities. Potential problems hindering the clinical use of curcumin are its low potency and poor absorption characteristics (12). The bioactivities and applications of curcumin have been well summarized elsewhere (13–17). Regardless, curcumin remains an ideal lead compound for the design of more effective analogs.

**Figure 1:**
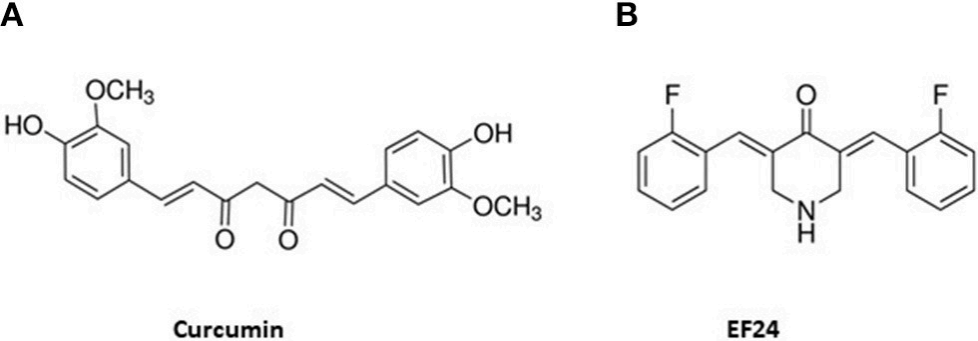
Chemical structures of curcumin **(A)** and EF24 **(B)**.

A promising curcumin analog, EF24, displays multiple potent bioactivities and increased bioavailability compared to curcumin. The chemical structures of curcumin and EF24 are shown in Figure 1. EF24 was first designed and synthesized by Adams et al. (18). The authors reported that EF24 induced cell cycle arrest and apoptosis via a redox-dependent mechanism in cancer cells (19). Later, EF24 was shown to have promising bioactivities, especially its anti-cancer activity in various solid tumors (18) and leukemia (20). Compared to the classical chemotherapy drug cisplatin, EF24 is more efficacious and less toxic (18). EF24 exerts its anti-cancer activity by inhibiting cancer cell proliferation or causing apoptosis via multiple pathways, such as inhibiting NF-κB (21), inhibiting HIF-1α activity (22), and regulating reactive oxygen species (ROS). In addition, EF24 shows promising anti-inflammatory (23–25) and anti-microbial activities (26). To improve the potency and bioavailability, new analogs were developed based on the structure of EF24. Here we will focus on summarizing in detail the known bioactivities and mechanisms of action of EF24 and briefly touch on the new derivatives in this review.

## Biological Activities and Mechanisms of Action of EF24

### Anti-cancer Activities

In 2004, Adams et al. synthesized and screened a series of curcumin analogs, among which EF24 demonstrated a high degree of cytotoxicity to cancer cells, showing higher potency than the commonly used chemotherapeutic drug cisplatin in inhibiting tumor cell growth (18). Additionally, EF24 was found to be the most potent anti-angiogenic compound among the analogs (almost as potent as the anti-angiogenic drug TNP-470) (18). *In vivo* studies showed that EF24 can effectively inhibit breast tumor growth with little toxicity in a mouse xenograft model (18), demonstrating the promise of EF24 as a chemotherapeutic agent for the first time. However, this study only revealed a preliminary and superficial knowledge of the mechanism for its anti-cancer effect (RNA/DNA antimetabolite) through a COMPARE analysis (18). In the next year, the authors reported that EF24 induced cell cycle arrest and apoptosis via a redox-dependent mechanism in human breast and prostate cancer cell lines (19). Evidence mainly came from the casepase-3 activation, phosphatidylserine externalization, depolarization of mitochondrial membrane potential, induction of ROS, and the inhibition of glutathione (GSH) (19).

Later, EF24 was found to have wide-spectrum anti-cancer activity. It is able to inhibit the proliferation of human cisplatin-resistant ovarian cancer cells via G2/M phase cell cycle arrest and increased G2/M checkpoint proteins (p53, p21) (27). In addition, EF24 can cause apoptosis in cisplatin-resistant cells by activating phosphorylated PTEN, which subsequently inhibited Akt and MDM2, enhanced p53 levels and finally induced cell cycle arrest and apoptosis (27). Another study on ovarian carcinoma showed that EF24 time- and dose-dependently suppressed the growth and synergized with cisplatin to induce apoptosis (28). In 2008, Subramaniam et al. reported that individual use of EF24 induced caspase-mediated apoptosis and inhibited the growth of colon cancer tumor xenografts (29). Combination of EF24 with other chemotherapy drugs also showed an impressive role in suppressing colon cancer growth (30). Accumulating evidence suggests EF24 to be active in cell and/or tumor models of numerous cancer types. For example, in *in-vitro* experiments, it is effective in inhibiting osteogenic sarcoma cells (31), malignant pleural mesothelioma cells (32), progressive medullary thyroid cancer cells (33), human pancreatic cancer cells (34) and leukemia/lymphoma cells (20, 35). Whether used alone or in combination with other agents, EF24 displays great potential as an anti-cancer therapeutic.

### Anti-cancer Mechanisms of EF24

#### Inhibition of NF-κB Signaling

Most studies suggest that EF24 impairs cell growth by inducing cell cycle arrest followed by induction of apoptosis, which is accompanied by caspase-3 activation. However, the cell signaling pathway mediating the EF24 effect was not elucidated until 2008 when Kasinski et al. first revealed that EF24 induced cell apoptosis via suppressing NF-κB signaling pathway through direct action on IkB kinase (IKK) (21). NF-κB regulates a wide variety of genes involved in cell proliferation, differentiation, cell cycle control (36), oncogenic activation (37) and metastasis (38). EF24 can inhibit the catalytic activity of IKK protein complex, which blocks IκB phosphorylation and subsequent degradation, and finally prevents the nuclear translocation of p65 subunit of NF-κB. The study provides a molecular explanation for the superior activity of EF24 over curcumin (with a potency about 10 times higher than that of curcumin). Other groups further verified the involvement of EF24 in inhibiting NF-κB signaling pathway in cancer (39–42). EF24 robustly conferred radiation-induced cell death mainly by inhibiting radiation-induced NF-κB signaling in breast cancer (43). In the same year, the same group found that EF24 can suppress the radiation-induced NF-κB-DNA binding activity/promoter activation in genetically varied human neuroblastoma (44). Inhibition of the NF-κB signaling extends the therapeutic application of EF24 to other NF-κB-dependent diseases, such as inflammatory diseases (described below).

#### Regulation of Fanconi Anemia (FA) and MAPK Pathways

By targeting IKK in the NF-κB pathway, EF24 was also able to inhibit the Fanconi anemia (FA) pathway, a multigene DNA damage response network implicated in the repair of DNA lesions. In HeLa cells, nanomolar concentrations of EF24 inhibited hydroxyurea (HU)-induced Fanconi anemia D2 ubiquitination (FANCD2-Ub) and foci in a cell-cycle independent manner (45). Beside the FA pathway, several other signaling pathways interplay with NF-κB, one of which is the mitogen-activated protein kinase (MAPK) pathway (46, 47). Given the substantial role of EF24 in inhibiting NF-κB, scientists started to notice its role in regulating MAPK pathway. Thomas et al. reported that EF24 drastically induced the upregulation of three major MAPK pathways mediated by ERK, JNK, and p38 (48). On the contrary, Lin et al. reported that EF24 exerts its anti-tumor activity in oral squamous cell carcinoma via deactivation of the MAPK/ERK signaling pathway (49). Therefore, the effect of EF24 on the MAPK pathway is still under debate and needs to be verified in more cancer types in the future.

#### Regulation of HIF-1α Expression

Another important role of EF24 is to regulate HIF-1α expression which is closely associated with the outcome of chemotherapy in cancer treatment. For example, Liang et al. reported that sorafenib therapy could induce drug resistance in the treatment of hepatocellular carcinoma, in which HIF-1α plays an important role (50). Anti-angiogenic effects of sustained sorafenib therapy caused intratumor hypoxia, which induced HIF-1α and protected cancer cells from sorafenib treatment. EF24 can inhibit HIF-1α by sequestering it in cytoplasm and can promote its degradation by upregulating Von Hippel-Lindau tumor suppressor (VHL). Combination of EF24 and sorafenib showed synergistic effects against metastasis both *in vivo* and *in vitro* (50), providing compelling evidence for the potential of clinical application of EF24. Similar to EF24, the parent compound curcumin can also regulate HIF-1α expression (51), albeit through distinct mechanisms. Curcumin inhibited HIF-1α gene transcription, while EF24 exerted the activity by inhibiting HIF-1α post-transcriptionally (22).

#### Regulation of ROS Production

The oncogenic factor HIF-1α is implicated in regulating aerobic glycolysis and the expression of Glut1 (52). Since, EF24 was able to inhibit HIF-1α activity, it likely affects glucose metabolism, thereby regulating cancer cell survival. Actually, unlike normal cells which produce energy through mitochondrial oxidative phosphorylation, most cancer cells produce their energy predominantly through a high rate of glycolysis followed by lactic acid fermentation, even in the presence of abundant oxygen, which is termed the Warburg effect (53). Although less efficient than oxidative phosphorylation in terms of ATP production, aerobic glycolysis generates additional metabolites that may help cancer cells to proliferate and growth. EF24 has been reported to block glucose uptake and the rate of glycolysis, and thereby inhibits cell migration and invasion in ovarian cancer cells (54). Another notable anti-tumor mechanism of EF24 is regulation of ROS production. Elevated oxidative status has been found in many types of cancer cells, which contributes to carcinogenesis (55–57). In 2014, Roy et al. reported that EF24 could protect protein disulfide isomerase (PDI), an important endoplasmic reticulum-resident oxidoreductase chaperone, from ROS-induced damage (58). In human ovarian cancer cells, EF24 suppressed ROS generation and activated antioxidant response element (ARE)-dependent gene transcription (28). Similarly, EF24 suppressed the level of superoxide in combination with SN38 in cancerous tissue (30). On the contrary, some other studies found that EF24 induces ROS production. The authors who first designed and synthesized EF24 observed that it was able to induce ROS production in MDA-MB-231 human breast cancer cells and DU-145 human prostate cancer cells (19). Similarly, EF24 was reported to induce ROS production in gastric cancer cells (59), and show synergistic anti-tumor activity with rapamycin (60) or Akt inhibitor (61). The same group reported a similar effect of EF24 on inducing ROS in human colon cancer lines (HCT-116 and SW-620 cells), but moderate effects in HT-29 cells (62). Therefore, it seems that the role of EF24 in ROS induction may be cell type-dependent, and more evidence is needed to clarify these contradictory results. Interestingly, Skoupa et al. recently concluded that apoptosis induced by the EF-24 is not mediated by oxidative stress-related in human leukemia cells (20). A redox-dependent-mediated mechanism only marginally contributes to the EF24-induced apoptosis in K562 cells. Therefore, the effects of EF24 on ROS and ROS-mediated apoptosis in cancer cells remains to be confirmed in the further studies.

#### Regulation of miRNA and miRNA Target Genes

EF24 was also found to target microRNAs to regulate the tumor progress. MicroRNAs (miRNAs) are short non-coding RNAs that post-transcriptionally regulate gene expression. Dysregulation of miRNA expression in cancer has been well-established (63). Yang et al. reported that EF24 was able to downregulate miR-21 and thereby enhance the expression of its target genes PTEN and PDCD4, which inhibit tumor growth and metastasis (64). Another group reported that EF24 suppressed melanoma metastasis by upregulating miR-33b and concomitantly reducing HMGA2 expression (65). The role of EF24 in regulating miRNAs provides a novel mechanism for its anti-cancer function.

#### Comparison of EF24 to Curcumin in Anti-cancer Activity and Bioavailability

Since the development of EF24, scientists have compared its activity with its parent compound curcumin. Most of the studies were focused on the anti-cancer activity. Adams et al. synthesized a series of curcumin analogs including EF24, and submitted them to the NCI anti-cancer cell line screen. The results showed that EF24 was effective against all of the cell lines. The mean panel GI_50_ (concentration at which the drug inhibits tumor cell growth by 50%) was 10-fold better than curcumin and cisplatin (18). The authors also submitted the analogs to an *in vitro* anti-angiogenesis screen and revealed that EF24 was more active than curcumin in the assay. Their *in vivo* experiment suggested that EF24 was well tolerated by mice and much safer than the chemotherapy drug cisplatin (18). Later, Subramaniam et al. further compared the potency of EF24 to curcumin in gastrointestinal cancer cells and demonstrated that EF24 was more potent than curcumin. For example, 1 μmol/L of EF24 significantly suppressed proliferation and colony formation of the colon and gastric cancer cell lines while at the same dose of curcumin had no effect (29). Consistently, EF24 exhibited IC_50_ values 10 to 20 times lower than that of curcumin in multiple cancer cell lines, including lung, ovarian, cervical, breast, prostate cancer cells and cholangiocarcinoma cells (21, 22, 28, 40, 48). Similarly, in human osteogenic sarcoma cells (Saos2), EF24 was 3-fold more potent than curcumin (31). This evidence collectively suggests that EF24 displays much more potent anti-cancer activity than its parent compound *in vitro*. Although EF24 shared many anti-cancer mechanisms with its parent compound curcumin, such as inhibiting NF-κB and HIF-1α, it exerts its effects in different ways, for example, in how it regulates HIF-1α activity (described above). In addition, curcumin efficiently inhibited proteasome activity. By contrast, EF24 was 20-fold less active than curcumin for proteasome inhibition (45). Likewise, curcumin can regulate the STAT3, while EF24 has no effect on STAT activation (64).

The *in vivo* activity of a compound relies on the bioavailability of the compound at the site of the tumor. Dietary curcumin is poorly absorbed through the intestinal tract, therefore curcumin does not have a therapeutic effect at low doses (29). By contrast, EF-24 has higher oral bioavailability (60%) in mice (66), explaining to some extent the improved *in vivo* activity of EF24 compared to curcumin.

In addition to the improvement of anti-cancer activity, EF24 shows low toxicity to normal cells. EF24 has been shown to induce apoptosis in cancer cells and inhibit the growth of human breast tumors in a mouse xenograft model but showed low toxicity (18). Subramaniam et al. reported that EF24 inhibited intestinal cancer cell proliferation, but did not affect the proliferation of normal mouse embryonic fibroblasts cells, suggesting that EF24 is not toxic to normal cells (29). Similarly, EF24 inhibits tumor growth in human cholangiocarcinoma while displaying low toxicity levels. As a sensitizer, co-treatment of EF24 and rapamycin selectively enhances the cytotoxicity in gastric cancer cells but not in normal cells (60). Above all, multiple molecular targets, wide-spectrum potency, enhanced bioavailability as well as low toxicity to normal cells confer EF24 a series of advantages in clinical applications.

### Anti-inflammation Activity

Inflammation serves as a common mechanism of various diseases including cancer (67), cardiovascular and neurodegenerative diseases (68, 69), diabetes (70), and certain neuropsychiatric disorders (71). The NF-κB pathway has been identified to mediate inflammation and therefore is considered as a critical target for development and discovery of drugs for these diseases (72, 73). Curcumin has been discovered as a blocker of NF-κB (74, 75). As the curcumin analog, EF24 was shown to inhibit LPS-induced pro-inflammatory cytokine mRNA expression and impair LPS-induced NF-κB nuclear translocation (23). Vilekar et al. reported that EF24 can reduce the expression of LPS-induced MHC class II, CD80 and CD86 molecules, as well as reduce NF-kB activity and TNF-α secretion (24). Similarly, EF24 was shown to suppress the LPS-induced TLR4 and IL-1R1 expression in dendritic cells (25), which initiate pro-inflammatory signaling upon ligand binding.

EF24 was also shown to reduce the expression of aquaporin-1, a gene that may be involved in the regulation of immune response in sepsis (76). Stimulation of polymorphonuclear granulocytes by LPS led to increased expression of aquaporin-1 *in vitro*, which could be abrogated by EF-24. Tissue inflammation led by hemorrhage and aggravated by reperfusion often causes irreversible organ damage. Given the significant inhibitory effect on NF-κB, EF24 may play a positive role in reducing the hemorrhage-induced inflammation and symptoms. Yadav et al. revealed that EF24 can prevent the inflammatory status in rats subjected to 50% hemorrhage, preserve the pulmonary histology, and improve the survival of hemorrhaged rats (77, 78). These effects were associated with the inhibition of NF-κB and IL-6R signaling, suggesting EF24 as a promising protective agent in hemorrhage-induced inflammation. The same group found that EF24 treatment suppressed pro-inflammatory signaling in liver tissue and improved liver functional markers in hemorrhagic shock (79). They also evaluated the effect of EF24 on intestinal barrier dysfunction in hypovolemic shock, and showed EF24 to attenuate hypovolemic gut pathology and protect barrier function by restoring the status of tight junction proteins (80). Because most chronic diseases are mediated through dysregulated inflammation, EF24 has potential use in the prevention of these diseases.

### Antibacterial Activity

Curcumin is well known for its antimicrobial properties, which has been summarized (81). However, as mentioned above, it has very poor bioavailability due to poor absorption in the intestinal tract and *in vivo* metabolism by the non-cytochrome P450 pathway. EF24 and its derived compounds are of considerable interest because of enhanced bioavailability and potency compared to that of curcumin (described above). In 2013, Vilekar et al. evaluated the antibacterial activity of EF24 and revealed it to suppress bacterial growth without affecting the bacterial uptake or localization in the dendritic cells (26). Even though the antibacterial potency of EF24 is much lower than the traditionally used antibiotics, it can potentially be applied as adjunct or chemopreventive agents in critical scenarios. With an aim of investigating cellular and molecular targets, Cocorocchio et al. employed *Dictyostelium discoideum* mutants and successfully identified the protein phosphatase 2A regulatory subunit PsrA and the presenilin 1 ortholog PsenB that were partially involved in the effect of curcumin and EF24 (82). Recently, Ramayanti et al. investigated the potential of curcumin and its analogs to trigger the Epstein-Barr virus (EBV) lytic cycle in nasopharyngeal (NPC) and gastric carcinoma cells. They found that EF24 showed high lytic inducing activity and enhanced the cytolytic virus activation (83). EF24 therefore may serve as a good adjuvant in cytolytic virus activation treatment.

### Neuroprotective Activity

Reports on the neuroprotective role of EF24 are very limited. One is related to the nitrosative stress that is causal in a select sporadic variant of Parkinson's (PD) and Alzheimer's (AD) diseases. Increased nitric oxide (NO) can disrupt the redox activity of protein-disulfide isomerase, a key chaperone in the endoplasmic reticulum by S-nitroso modification of its redox-active cysteines (84, 85). Curcumin has been demonstrated to scavenge nitric oxide (NO) species from model NOx donors (86). Similarly, EF24 exerts neuroprotective effects by ameliorating nitrosative stress-linked damage to protein-disulfide isomerase (PDI) and the associated onset of PD and AD (87).

### Progress on Improving Bioactivity and Bioavailability of EF24

#### New Derivatives Improved Bioactivity of EF24

To further improve the anti-cancer activity, some groups have moved forward to design and synthesize new analogs of EF24. In 2012, Lagisetty et al. synthesized a hydrazinonicotinic acid conjugate using an amine derivative of EF24. The derivative has improved aqueous solubility compared to EF24, and showed significant anti-tumor effects (88). Wu et al. synthesized and purified 20 EF24 analogs, from which they identified one to have greater activity than EF24, showing potent anti-migration and anti-proliferative activity against A549 cells (89). Xie et al. designed and synthesized four series of EF24 analogs, from which they found one displaying excellent inhibition of both IKKβ activity and pancreatic cancer (PC) development and progression (90). A group from Germany synthesized 14 EF24 analogs and revealed that they have promising anti-proliferative activity against eight cancer cell lines with low IC_50_ values, and showed superior anti-angiogenic and vascular-disruptive effects (91). Last year, a research group from China synthesized a series of EF24 analogs and identified one to have much greater inhibitory activity against IKKβ and to induce apoptosis and cell cycle arrest in multiple cancer cell lines (92). Recently, a series of EF24 analogs were synthesized and screened, resulting in one with good potency and selectivity in killing cancer cells (93).

#### New Drug Delivery Systems (DDS) to Improve the Bioavailability of EF24

Improving the bioavailability of chemotherapy drugs can greatly increase the therapeutic effect while reducing the side effects. To achieve this aim, specific drug delivery systems (DDS) have been investigated. For example, scientists observed that the tissue factor is aberrantly and abundantly expressed in many cancer cells (94, 95), and based on this expression, the group that first designed and synthesize EF24 later proposed a new drug delivery system that associated with tissue factor on the surface of cancer cells, releasing the cytotoxic agent into the cytoplasm. This system displayed a greater effect than EF24 alone in human breast and melanoma cell lines (96). Later, it was reported that the conjugation of EF24 with coagulation factor VIIa induced apoptosis in tumor cells and significantly reduced tumor size in human breast cancer xenografts in athymic nude mice. The targeted drug delivery system has the potential to enhance therapeutic efficacy, while reducing toxic side effects (97). Although EF24 has shown promising *in vitro* therapeutic efficacy in various human cancer cells, increasing water solubility and systemic bioavailability will be beneficial for its clinical applications. Agashe et al. designed EF24-liposomes and showed their anti-proliferative activity to be superior to that of EF24 alone (98). Bisht et al. designed nano-encapsulation of EF24 into pegylated liposomes (Lipo-EF24), evaluated the particles in pancreatic cancer models, and observed good therapeutic efficacy and favorable toxicity profile (99), which provide evidence for development of future combinatorial therapeutic regimens against pancreatic cancer.

### Conclusions and Perspectives

**Figure 2:**
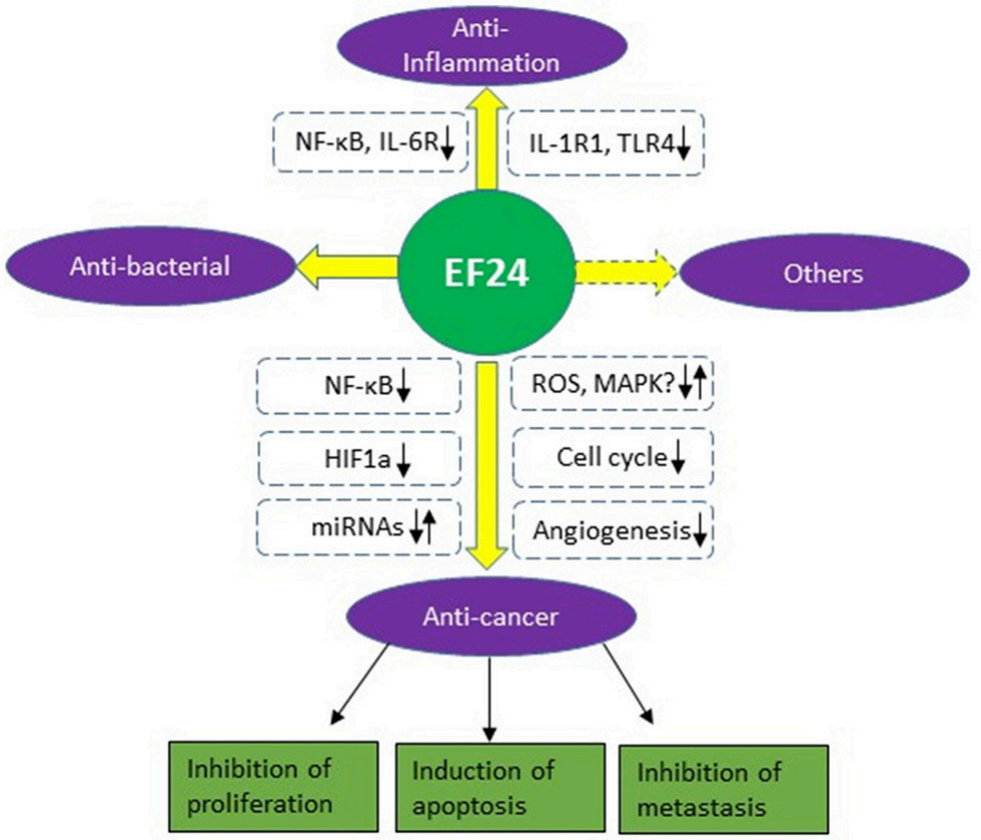
Biological activities and mechanisms of EF24.

This review gives a brief summary of the biological and pharmacological activities of EF24, a novel analog of curcumin (Figure 2). Enhanced bioavailability and potency makes it promising as a therapeutic compound alone or combination use with other agents. EF24 has been found to suppress tumor growth by inducing cell cycle arrest or apoptosis in many cancer types. It also shows good anti-inflammatory and anti-bacterial activity. The main mechanisms of action for EF24 include inhibition of the NF-κB pathway and HIF-1α protein and regulation of the MAPK pathway and ROS production. The latter two may be cancer/cell type-dependent and need to be confirmed in future studies.

Natural products usually have biological molecular targets, which confers them multiple bio-activities. Exploring and understanding the targets will provide insights into the mechanisms of action of a natural product and help to improve its efficiency and lower side effects in clinical applications. Based on the known activities of curcumin, we can expect that EF24 may have additional unexplored bioactivities and molecular targets. For example, like some triterpenoid compounds (100, 101), curcumin plays an important role in regulating energy metabolism (102), while application of EF24 in metabolic diseases needs to be determined. In addition, because current therapeutic effects of EF24 are based on cell culture and animal studies, clinical trials are needed to fully verify its therapeutic potential. It is promising that we bring this natural product to the forefront of therapeutic agents for the treatment of human diseases.

## Author Contributions

YH: Manuscript writing and figure preparation; WL, GH, HS, and QK: Literature search and manuscript editing.

### Conflict of Interest Statement

The authors declare that the research was conducted in the absence of any commercial or financial relationships that could be construed as a potential conflict of interest.
